# *Agaricus bisporus* Mannose-Binding Protein Stimulates the Innate Immune Cells

**DOI:** 10.34172/apb.43767

**Published:** 2024-12-13

**Authors:** Wangsa Tirta Ismaya, Agung Heru Karsono, Olivia Mayasari Tandrasasmita, Raymond Rubianto Tjandrawinata, Heni Rachmawati

**Affiliations:** ^1^Dexa Laboratories of Biomolecular Sciences, Industri Selatan V Blok PP-7, Jababeka II Industrial Estate, Cikarang 17559, West Java, Indonesia.; ^2^Faculty of Biotechnology, Atma Jaya Catholic University of Indonesia, Jl. Raya Cisauk-Lapan 10, Tangerang 15345, Banten, Indonesia.; ^3^Research Group of Pharmaceutics, School of Pharmacy, Bandung Institute of Technology, Ganesa 10, Bandung 40132, West Java, Indonesia.; ^4^Research Centre for Nanosciences and Nanotechnology, Bandung Institute of Technology, Ganesa 10, Bandung 40132, West Java, Indonesia.

**Keywords:** Breast cancer, Cellular immunity, Innate immune system, Lectin, Macrophage, Mannose-binding protein

## Abstract

**Purpose:**

A lectin-like protein from the mushroom *Agaricus bisporus* has been shown to slightly increase the proliferation of RAW 264.7 cells. Following its identification as a mannose-binding lectin, henceforth called *A. bisporus* mannose-binding protein (Abmb), the protein is hypothesized to stimulate the innate immune cells response. The present work was aimed to substantiate that hypothesis. Furthermore, this study complements Abmb exploration as a potential agent for anti-breast cancer, which its treatment is hampered with compromised immunity of patient receiving chemotherapy.

**Methods:**

Abmb’s effect on the phagocytic activity of the macrophage was measured with FACS. Nitric oxide (NO) production was checked using Griess test while expression of the cytokines in the RAW 264.7 cells was analysed at gene and protein level using polymerase chain reaction (PCR) and FACS, respectively. Abmb’s effect on the expression of surface markers of the human immune cells in the peripheral blood mononuclear cells (PBMCs) was checked with specific antibodies for targeted cluster differentiation (CD) and analysed using FACS.

**Results:**

Abmb increased the phagocytic activity of the macrophage and NO production. Abmb increased the expression of cytokines *i.e.* tumour necrosis factor (TNF)-α, interleukin (IL)-6, and IL-10. With the PBMCs, Abmb activated dendritic and natural killer (NK) cells, but not the B- or T-cells.

**Conclusion:**

Abmb increased the activity of the macrophage cells and activated the immune cells that are related to the innate immune system, particularly the cellular immunity.

## Introduction

 The light subunit of mushroom *Agaricus bisporus* tyrosinase (LSMT) was found to slightly enhance the proliferation of RAW 264.7 cells (macrophage).^1^ Recently, LSMT was identified to specifically bind mannose and mannitol, but not galactose, glucose, nor sorbitol.^2^ Hence, LSMT was renamed to Abmb (*A. bisporus* mannose-binding protein). That recent finding may explain Abmb effect on the macrophage because mannose-binding protein (MBP) is a key player in the innate immune system.^3^ Most of MBP structurally has the collectin type lectin (CTL) fold that contains calcium, which is required to bind mannosyl-sugars.^4^ MBP with non-CTL structure can induce the immune cell response as demonstrated by concanavalin. Concanavalin has a β-prism fold, binds glucose, and requires metal ions for sugar-binding.^5^ Abmb structure contains no calcium and belongs to the ricin B-like type lectin (RTL).^6,7^ RTL exclusively consists of glucose-/galactose-binding protein and requires no metal cofactor to bind its sugar target.^8^ RTL could also influence the immune cells response as demonstrated by sMTL-13 that increases IFN-γ production by the peripheral blood mononuclear cells (PBMCs) in the blood serum of active tuberculosis patients.^9^ Furthermore, the residues responsible for sugar recognition in RTL are equally present in Abmb.^10^ Hence, Abmb effect on the immune cells must be substantiated.

 Abmb does not evoke the generation of IgG in Swiss Webster and Balb/c mouse even after 12 weeks of weekly administration period. Histopathological evaluation further suggests that Abmb does not induce organ damage.^11,12^ Thus, Abmb is not immunogenic or toxic. That early study also indicates that Abmb likely has no effect on the adaptive immune system. One of Abmb’s closest structural homologs (thus an RTL), HA-33 from the botulinum toxin complex, has been reported to evoke the generation of IgG in mice.^13^ However, HA-33 is a galactose-binding lectin. The other Abmb’s closest structural homolog is the mushroom *Clitocybe nebularis* lectin (CNL), which induces the maturation and activation of dendritic cells (DCs).^14^ CNL’s effect on macrophage has not been reported. Like HA-33, CNL is also a galactose-binding protein. This situation indicates that Abmb might exert its activity differently to its structural homologs.

 Lectin has been strongly linked to stimulation and/or activation of the innate immune system *via* the complementary lectin pathway, which is based on glycan/sugar recognition upon interaction with the receptor on the cell surface.^3^ Hence, the type of glycan dictates the pathway to activate.^15^ Phagocytic activity of the macrophage is the most common effect induced by lectin, but the reaction cascade in the cells depends on which receptor is activated.^16^ In this instance, CTL and RTL exert their activity differently due to differences in their sugar targeting.^4,8^ As for Abmb, exploring its effect on the immune cells becomes challenging.

 Here, the Abmb effect on the macrophage was evaluated in terms of phagocytic activity, production of nitric oxide (NO), and cytokines *i.e.* tumour necrosis factor (TNF)-α, interleukin-6 (IL-6), IL-10. Abmb effect on human immunity was studied through the expression of surface markers for T-, B-, natural killer (NK), dendritic, and monocyte/macrophage (M/M) cells in PBMC. The present study provides more hints for further works to elucidate the pathway upon stimulation of the immune cells by Abmb.

## Material and Methods

###  Materials

 The chemicals were purchased from Merck (Darmstadt, Germany) except when specifically mentioned. Abmb was prepared according to a previous report.^17^ Details of Abmb preparation is provided in Supplementary file 1. The RAW 264.7 cells were from ATCC, and the tissue culture media was from Gibco (Grand Island, NY, USA). PBMC were isolated from human blood, obtained from donors with their consent. Reagents for FACS analysis were from BD Biosciences (San Jose, CA, USA).

###  Abmb effect on the macrophage cells 

 RAW 264.7 cells were grown in a 96-well plate (Thermo Fischer Scientific, Singapore) according to the previous report.^1^ Abmb was added to the cells at a final concentration of 0.18-1.41 μM. The phagocytosis assay was performed as described by Sharma with a minor modification^18^ at a final Abmb concentration of 0.35-1.41 μM. Briefly, the fluorescence beads were opsonized with FBS for 1 hour before use. The beads were incubated with Abmb for 2 hours, added to the macrophage cells, and incubated for another 2 hours. The fluorescence signal of the engulfed beads was measured in a FACSCalibur system (BD Biosciences, San Jose, CA, USA) and were analysed with the Cell Quest program. The amount of NO in the cell media was measured using Griess reagent (Promega, Madison, WI, USA). The cytokine gene expression analysis was done from the total RNA sample collected at 2, 18, 24 and 48 hours of incubation. RNA was isolated using RiboEx (GeneAll, Seoul, Korea) and cDNA was generated from total RNA sample using ReverTra Ace (Toyobo, Osaka, Japan). Amplification was done using GoTaq (Promega, Madison, WI, USA) and performed in a T3000 Thermocycler (Biometra, Göttingen, German). Images were captured using Chemi-Doc (BioRad, Singapore). The relative levels of target gene mRNA expression were normalized to actin as the internal control. Gene expression was evaluated using gene-specific primers for TNF-α, IL-6, IL-10, and actin (marker) (Table S1, Supplementary file 1). The cytokine analysis at the mRNA level was performed using a cytometric bead array mouse inflammation kit (BD Biosciences, San Jose, CA, USA) following the manufacturer’s instruction. Lipopolysaccharide (LPS) (at ~0.01 μM; ~1 μg/mL, assuming the size is ~ 100 kDa) was employed as the positive control to illustrate when the immune system is activated.

###  Cell surface marker analysis

 The analysis used specific antibodies for the targeted cluster differentiation (CD) in the whole blood sample. After 24 hours of treatment with Abmb (added concentration of 1.41 μM), PBMC cells were collected by centrifugation at 4 °C, suspended in PBS, and aliquot in 10-mL sample tubes. PBS was used as the control. CD was cross-reacted for 30 minutes and then the cells were then centrifuged, washed and suspended in PBS. Conjugate of CD-antibody was measured with a FACSCalibur system (BD Biosciences, San Jose, CA, USA) and analysed with the Cell Quest program. The CD antibodies were purchased from Invitrogen (Thermo Fischer Scientific, Singapore), BioLegend (San Diego, CA, USA) and BD Bioscience (San Jose, CA, USA).

###  Statistical analysis

 Statistics were determined by unpaired t-test analysis using QuickCalcs (GraphPad Software, Boston, MA, USA). Values are expressed as mean ± standard deviation (SD) derived from at least three independent experiments with *P* < 0.05 considered as significant.

## Results

###  Abmb effect on proliferation of macrophage

**Figure 1:**
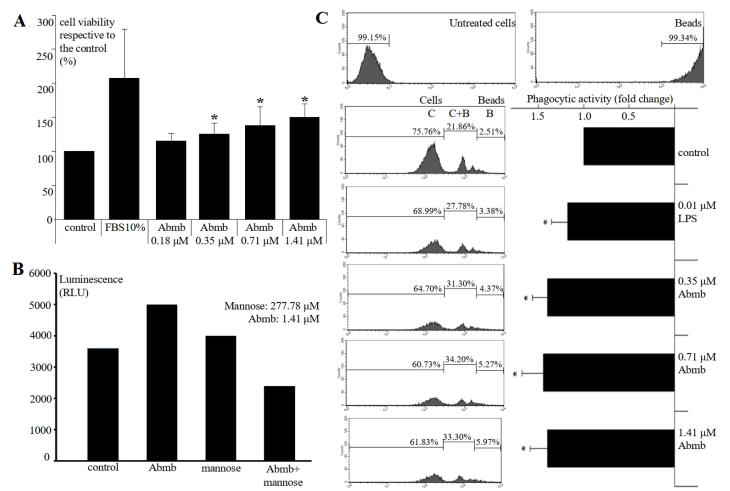
(A) Proliferation of the RAW 264.7 cells upon 24-hours treatment with Abmb at an increasing concentration. (B) The fluorescence values of cells treated with Abmb, mannose, and their combination relative to the control. (C) Phagocytic activity of Abmb at increasing concentrations on RAW 264.7 cells. The values are expressed as mean ± SD (*P < 0.05, compared to the control)

 Previously, the effect of Abmb on macrophage was reported to become obvious at concentrations higher than 1.41 μM.^1^ In the present study, the effect was checked at lower Abmb concentrations to recognise the macrophage cells sensitivity towards the protein. The result shows that Abmb started to induce macrophage proliferation at 0.35 μM (Figure 1A). This concentration is lower than reported previously.^1^ Further, Abmb ability to increase the proliferation of macrophage was diminished when the protein was incubated with mannose prior to addition to the cells to block its sugar binding site (Figure 1B). Mannose itself has negligible effect on the proliferation, which is in agreement with the report of others.^19^ Similar result was observed upon testing with the MCF-7 cells, where Abmb also loss its ability to supress the proliferation of the breast cancer cells when the protein was pre-incubated with mannose.^2^ The loss of Abmb effect upon blocking with mannose provides a hint for the protein’s mechanism of action.

 Finally, Abmb slightly increased phagocytic activity of macrophage (Figure 1C) and increasing Abmb amount did not enhance phagocytic activity. Thus, Abmb appears to increase macrophage’s proliferation and phagocytic activity, but the effect is restrained. This result is also similar to Abmb effect to the breast cancer cells, where the protein only causes growth arrest at low concentrations as demonstrated with the MCF-7^1,2^ and MDA-MB-231 cells.^17^ MBP is widely known to bind specifically breast cancer cells, which are abundantly decorated by mannose-type glycans.^20,21^ Mannose and lectin interaction is also well known in regulation of immunity.^15^ This study provides further support for the relationship between immunity and cancer, in which cellular immunity response is able to counter internal infection and deal with cancerous cells.^22^

###  Abmb’s effect on production of NO and the cytokines

**Figure 2:**
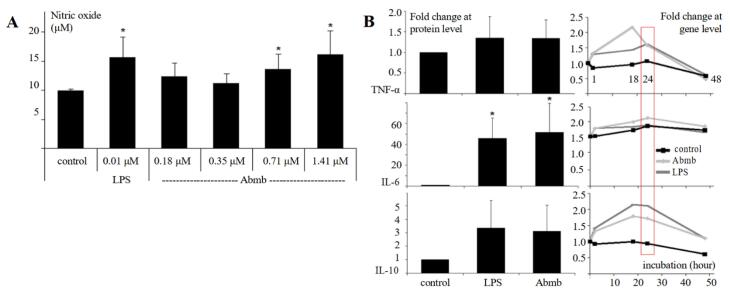
(A) Production level of NO in the absence and presence of Abmb at increasing concentration. (B) Protein level of cytokines after 24 hours of Abmb addition; at gene level is in time course (the red box indicates the gene level at 24th hours correspond to the protein level). The figure legends are indicated next to the vertical and bottom horizontal axis. The values are expressed as mean ± SD (*P < 0.05, compared to the control)

 Abmb increased NO production in the macrophage cells, and the increase was related to Abmb concentration (Figure 2A). The effect of Abmb addition at 0.18 and 0.35 μM to NO production was not significant and the value was lower than that of LPS, which was employed as the control. This result indicates that the effect of Abmb was clean from LPS because the effect should increase as the concentration increased (due to dilution factor of the sample). LPS at very low concentration has been reported to induce chemokines and cytokines.^23^ Further, this result also correlates with the output of the screening (see previous part). An increased in the NO production level is indicative for the stimulation of cytokine production, particularly by TNF-α. Thus, production of the cytokines upon addition of Abmb was further investigated.

 Abmb increased the protein level of TNF-α, IL-6, and IL-10 after 24 hours (Figure 2B). However, the increase was only significant for IL-6 (~50-fold) while that of TNF-α and IL-10 was 1.5- and 3-fold, respectively (Figure 2B). Thus, Abmb induces pro-inflammatory reaction and the subsequent counterbalance. This finding agrees with the report that RTL increases the production of NO and the expression of TNF-α and IL-6 genes.^24,25^ Further, although the cytokine expression at protein level upon induction with Abmb was similar to that of LPS, the expression profile at gene level was different. At the gene level, the expression level of TNF-α, IL-6, and IL-10 upon addition of Abmb increased already in the first hour. With TNF-α, the level rapidly increased to more than double up to 18th hours and then returned to basal. This return is normal as the cell has responded to the stimulation.^26^ The return may also be related to the IL-10 expression, which appears to follow that of TNF-α. This counteractive action was reflected with their protein levels. Meanwhile, after its initial increase, IL-6 gene expression was relatively stagnant, but its protein level remained high. Thus, IL-6 appears not to be countered by IL-10, or its degradation mechanism was not active. Finally, the protein level of monocyte chemotactic protein (MCP)-1 was also doubled (data not shown), which further supported that the cellular immune cells response is active.

 RTL induces NO production and expression of TNF-α and IL-6 genes occurs *via* the TLR-4 pathway, as shown by the Ricin toxin-binding subunit B.^25^ The pathway is adopted upon maturation and activation of DC by CNL.^14^ MBL also interacts with TLR-4 to exert its activity.^27^ Thus, Abmb could adopt this pathway. However, the mechanistic action of Abmb is not yet clear because it can either binds a surface receptor that structurally contain mannosyl glycan, interact directly with a surface receptor, or compete with a surface receptor to bind the glycan on its cellular molecule target.

###  Abmb’s effect on the population of human innate immune cells

**Figure 3:**
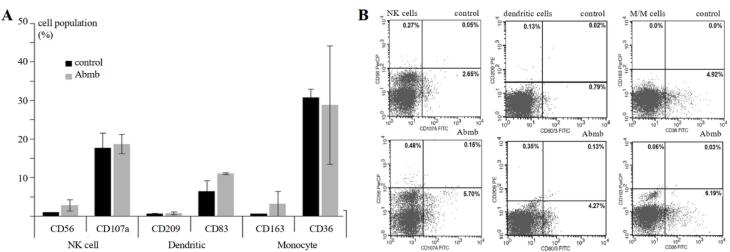
(A) Cell population of surface markers for NK cell, DC, and monocyte without or upon treatment with Abmb and (B) the fluorescence profile of the cells in FACS analysis

 Abmb effect on immune cells in PBMC was evaluated through detection of their specific CD markers, which are CD56 (NK cells), CD107 (activated NK cells), CD209 (monocyte-derived immature and mature DC), CD83 (mature DC), CD163 (M2 macrophage), and CD36 (Monocyte/Macrophage) after 24 hours of Abmb administration (Figure 3).

 Abmb slightly increased the cell population of CD56 but not CD107, which suggests that NK cells were activated, but not becoming cytotoxic. Abmb might have a regulatory function because NK cells action is restrained. CD107a is normally up regulated upon stimulation of the NK cells disregard the secreted cytokines.^28^ NK cells promote maturation and subsequent activation of DC through the secretion of TNF-α and IFN-γ that in turn releases IL-12 to activate NK cells.^29^ NK cells also destroy immature DC and hence it has been employed to discriminate the mature and immature DC.^30^

 Abmb increased the population of CD83 but not CD209. The result suggests that Abmb participates in maturation/activation of DC but not in DC differentiation from monocytes. CD83 is the marker for mature DC, which is considered as the link between innate and adaptive immunity for its ability to stimulate naïve T-cells.^31^ CD209 is the surface marker for monocyte-derived DC (immature and mature).^32^ Maturation of DC involves surface pattern recognition receptor, one of which is CD209 that specifically recognizes mannose. CD209 binds mannose at the positions C2-OH/C3-OH and the binding involves two glutamate and one asparagine residues, which is typical for CTL.^33^ The mannose-binding in Abmb likely includes two aspartate and one glutamine residues,^10^ thus similar to CD209. Based on this, Abmb and CD209 may compete for the binding of cellular molecule with a mannosyl ligand. Finally, Abmb did not change the level of CD163 and CD36, which are the surface markers for M2 macrophage^34^ and M/M,^35^ respectively. The latter set of CDs further suggest that monocyte was not differentiated into macrophages in the presence of Abmb.

 The above results show that Abmb could stimulate synergic action of NK cells and DC, which has been developed as a powerful strategy in anti-cancer immunity.^36^ The mechanism of Abmb action on macrophage and breast cancer cells is still elusive. Breast cancer cells could modify the surrounding microenvironment and thereby escape the immune system.^37^ Compromised immunity is a major issue in breast cancer therapy: the patients receiving adjuvant therapy with doxorubicin and cyclophosphamide are exposed to high risk of grade-3 infection due to the lower mannose-binding lectin 2 expression in their body.^38^ Supplementation of MBP during anticancer therapy is one of strategies in immunotherapy^39^ and Abmb could be developed for that purpose.

 Finally, Abmb effect is apparent to DC maturation and NK cell activation, which may lead to the activation of adaptive immunity. Further, additional testing showed that Abmb administration did not elevate the expression level of CD3 and CD25 (T-cells marker) and CD20 (B-cells marker) (data not shown). Thus, Abmb appears not to evoke the adaptive and only stimulate the innate immune system. This result concurs with Abmb non-immunogenic profile. The latter further suggests that Abmb does not stimulate humoral immunity because no antibody was generated, and the T cells were not activated. Abmb probably stimulates the non-adaptive cellular immunity response.

## Conclusion

 Abmb increased proliferation and phagocytic activity of the macrophage but in a restrained fashion. Similarly, Abmb also increased the expression of NO and TNF-α genes but the effect was restrained. This was further observed with the NK cells, which was activated but not becoming cytotoxic. Thus, Abmb could stimulate the immune cells response but not excessively to induce pro-inflammatory reaction. The present works support Abmb development as an immunotherapy agent. However, the observed phenomena could not yet determine the pathway adopted by Abmb to exert its activity: it could be as an RTL or CTL. At the moment, the study using glycan microarray to reveal Abmb’s target on the cell surface is in pursue.

## Competing Interests

 The authors declare no conflicts of interest.

## Ethical Approval

 No ethical issues to declare. No specific material, animal, or human subject used in this study.

## Supplementary Files

Supplementary file 1 contains Table S1 and Figure S1.
