# Supplementary material for: Agaricus bisporus Mannose-Binding Protein Stimulates the Innate Immune Cells
Source: Adv Pharm Bull. 2024 Dec 13;14(4):944–50. doi: 10.34172/apb.43767 (PMC11970490; doi:10.34172/apb.43767)
Supplement: Supplementary file 1 — contains Table S1 and Figure S1. [file apb-14-944-s001.pdf]

*Adv Pharm Bull*. 2024;14(4):944-S1
doi: 10.34172/apb.43767
https://apb.tbzmed.ac.ir

# Supplementary file 1

## Supplementary material S1: Production of Abmb

*Escherichia coli* BL21 (DE3) carrying the Abmb gene was grown for 12–14 hours at 37°C in LB medium containing ampicillin. Abmb expression was induced with 0.05 mM isopropyl β-D-1-thiogalactopyranoside (IPTG). The cells were collected by centrifugation at 4°C, 6,000 *g*, for 15 minutes, and subsequently washed with phosphate buffer saline (PBS; 137 mM NaCl, 2.7 mM KCl in 10 mM phosphate buffer, pH 7.4). The cells were resuspended in low-salt PBS (27.5 mM NaCl, 0.5 mM KCl in 10 mM phosphate buffer, pH 7.4) and then sonicated on ice (Misonix, QSonica, Newtown, CT-USA). The crude extract was clarified by centrifugation at 10,000 *g*, 4°C for 15 minutes and then loaded onto a Ni-NTA affinity column (Roche, Singapore). The pure $His_6$-tagged Abmb was recovered from the affinity column by elution with 200 mM imidazole in the low-salt PBS buffer. Finally, Abmb was cleaned on an Enrich™ SEC70 size exclusion column (BioRad, Singapore) using PBS buffer as the eluent. Purification steps were done with a NGC Scout-Plus chromatography system (BioRad, Singapore). Pure Abmb was stored at -20°C. Prior to use, the protein was thawed, centrifuged at 12,000 *g*, 4°C for 15 minutes, and sterilized over a 0.22 µm membrane filter. The protein concentration was estimated from its absorbance at 280 nm on a nanodrop 2000c spectrophotometer (ThermoFischer Scientific, Singapore).

The purification steps starting from the size exclusion column and protein stock preparation (extensive buffer exchange over several membrane filters) were done in water-for-injection (WFI) based PBS, which had been tested for endotoxin-free conditions. Prior to use, Abmb concentration was adjusted to 58.8 µM (~1 mg/ml, MW ~17 kDa) by dilution with the endotoxin free PBS.

The endotoxin level in Abmb stock solution was less than ~8 ng/mL or ~0.08 nM as measured with a Gel-clot assay (AccuMedi Solution, Zhanjiang, China). As the control of experiment, 0.01 µM LPS (or ~1 µg/mL, assuming MW ~ 100 kDa).

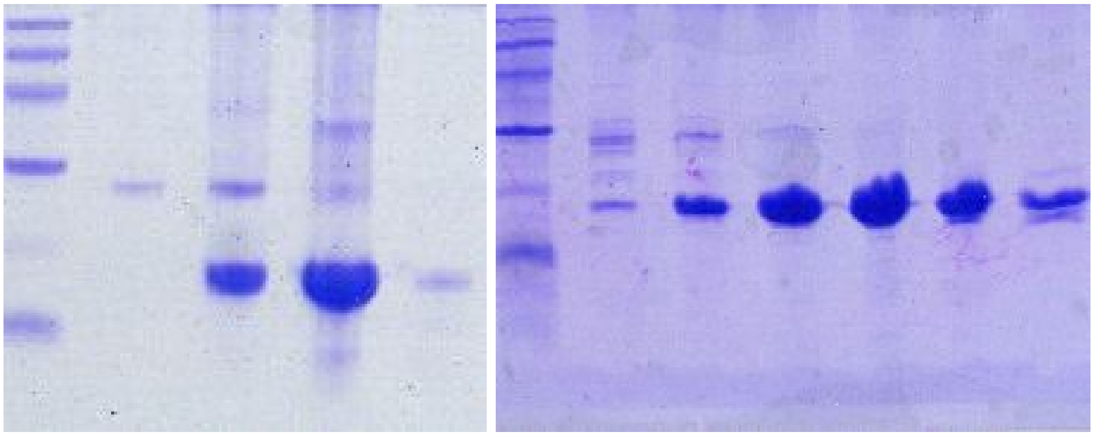

1 2 3 4 5 6 7 8 9 10 11 12

**Figure S1.** Purification of Abmb. Lane (1) and (6) are the molecular weight marker. The lanes (2), (3), (4) and (5) are the content of the eluted fractions from the Ni-NTA column during washing, early, main, and end of elution, respectively. The lane (7) is content of the fraction eluted in the dead volume of the SEC column, while (8-9) and (10-12) are of the early and main elution, respectively. Only fractions 10-12 were pooled, concentrated, and buffer exchanged for used in the experiments.

**Table S1.** List of primers

| Gene | Forward | Reverse |
|---|---|---|
| TNF-α | 5’-AGC-CCA-CGT-CGT-AGC-AAA-3’ | 5’-GGC-AGA-GAG-GAG-GTT-GAC-TT-3’ |

| | | |
|---|---|---|
| IL-6 | 5'-AGT-TGC-CTT-CTT-GGG-ACT-GA-3' | 5'-TCC-ACG-ATT-TCC-CAG-AGA-AC-3' |
| IL-10 | 5'-AAG-GGT-TAC-TTG-GGT-TGC-CA-3' | 5'-TTC-AGC-TCC-TCA-CCC-AGG-GA-3' |
| actin | 5'-ACC-CAC-ACT-GTG-CCC-ATC-TA-3' | 5'-CGA-AAC-CGC-TAC-TTG-CC-3' |
